# Public information needs and preferences on COVID-19: a cross-sectional study

**DOI:** 10.1186/s12889-023-15131-x

**Published:** 2023-02-27

**Authors:** Julia Lühnen, Thomas Frese, Wilfried Mau, Gabriele Meyer, Rafael Mikolajczyk, Matthias Richter, Jan Schildmann, Matthias C. Braunisch, Falk Fichtner, Christopher Holzmann-Littig, Peter Kranke, Maria Popp, Christian Schaaf, Christoph Schmaderer, Christian Seeber, Anne Werner, Marjo Wijnen-Meijer, Joerg J. Meerpohl, Anke Steckelberg, AP6 CEOsys, Astrid Viciano, Carina Benstöm, Georg Rüschemeyer, Holger Wormer, Jörg Wipplinger, Julia Krieger, Karolina Dahms, Kelly Ansems, Marcus Anhäuser, Patrick Meybohm, Stephanie Weibel

**Affiliations:** 1 Martin Luther University Halle-Wittenberg, Interdisciplinary Center for Health Sciences, Institute of Health and Nursing Science, Magdeburgerstraße 8, 06112 Halle (Saale), Germany; 2 Martin Luther University Halle-Wittenberg, Clinic for Internal Medicine I, Halle (Saale), Germany; 3 Martin Luther University Halle-Wittenberg, Interdisciplinary Center for Health Sciences, Institute of General Practice and Family Medicine, Halle (Saale), Germany; 4 Martin Luther University Halle-Wittenberg, Interdisciplinary Center for Health Sciences, Institute of Rehabilitation Medicine, Halle (Saale), Germany; 5 Martin Luther University Halle-Wittenberg, Interdisciplinary Center for Health Sciences; Institute of Medical Epidemiology, Biometrics and Informatics, Halle (Saale), Germany; 6 Martin Luther University Halle-Wittenberg; Interdisciplinary Center for Health Sciences; Institute of Medical Sociology, Halle (Saale), Germany; 7 Martin Luther University Halle-Wittenberg; Interdisciplinary Center for Health Sciences, Institute for History and Ethics of Medicine, Halle (Saale), Germany; 8 Technical University of Munich, School of Medicine, Klinikum rechts der Isar, Department of Nephrology, Munich, Germany; 9 Department of Anesthesiology and Intensive Care, University of Leipzig, Medical Center, Leipzig, Germany; 10 Technical University of Munich, School of Medicine, TUM Medical Education Center, Munich, Germany; 11 Department of Anaesthesiology, Intensive Care, Emergency and Pain Medicine, Faculty of Medicine, University of Wuerzburg, Wuerzburg, Germany; 12 Department of Medical Psychology and Medical Sociology, University of Leipzig, University Medical Center Leipzig, Leipzig, Germany; 13 Cochrane Germany Foundation, Cochrane Germany, Freiburg, Germany; 14 Medical Center & Faculty of Medicine, Institute for Evidence in Medicine, University of Freiburg, Freiburg, Germany

**Keywords:** Needs assessment, Consumer health information, Information dissemination, Information seeking behaviour, Pandemic

## Abstract

**Background:**

Right from the beginning of the SARS-CoV-2 pandemic the general public faced the challenge to find reliable and understandable information in the overwhelming flood of information. To enhance informed decision-making, evidence-based information should be provided.

Aim was to explore the general public’s information needs and preferences on COVID-19 as well as the barriers to accessing evidence-based information.

**Methods:**

We performed a cross-sectional study. Nine hundred twenty-seven panel members were invited to an online survey (12/2020-02/2021). The HeReCa-online-panel is installed at the Martin Luther University Halle-Wittenberg to assess regularly the general public’s view on health issues in five regions in Germany. The survey was set up in LimeSurvey, with nine items, multiple-choice and open-ended questions that allowed to gather qualitative data. Quantitative data were analysed descriptively and a content analysis was carried out to categorise the qualitative data.

**Results:**

Six hundred thirty-six panel members provided data; mean age 52 years, 56.2% female, and 64.9% with higher education qualifications. Asked about relevant topics related to COVID-19, most participants selected vaccination (63.8%), infection control (52%), and long-term effects (47.8%). The following 11 categories were derived from the qualitative analysis representing the topics of interest: vaccination, infection control, long-term effects, therapies, test methods, mental health, symptoms, structures for pandemic control, infrastructure in health care, research. Participants preferred traditional media (TV 70.6%; radio 58.5%; newspaper 32.7%) to social media, but also used the internet as sources of information, becoming aware of new information on websites (28.5%) or via email/newsletter (20.1%). The knowledge question (Which European country is most affected by the SARS-CoV-2 pandemic?) was correctly answered by 7.5% of participants. The Robert Koch Institute (93.7%) and the World Health Organization (78%) were well known, while other organisations providing health information were rarely known (< 10%). Barriers to accessing trustworthy information were lack of time (30.7%), little experience (23.1%), uncertainty about how to get access (22.2%), complexity and difficulties in understanding (23.9%), and a lack of target group orientation (15,3%).

**Conclusions:**

There are extensive information needs regarding various aspects on COVID-19 among the general population. In addition, target-specific dissemination strategies are still needed to reach different groups.

**Supplementary Information:**

The online version contains supplementary material available at 10.1186/s12889-023-15131-x.

## Introduction

On 17th December 2020 the COVID-19 7-day incidence in Germany reached 179 cases per 100,000 inhabitants [1]. Several measures for pandemic control had already been implemented (e.g. close-down of restaurants, gyms, theatres and cinemas as well as travel and contact restrictions). On 16th December, schools and stores without goods for daily use had to close. On 18th February 2021, the 7-day incidence had fallen to 57 cases per 100,000 inhabitants. Vaccination against COVID-19 had started at the end of December and at this time, 3.6% of the German public was vaccinated at least once. A new test strategy with free tests to enable the reopening of schools and stores was discussed. The development of the pandemic and the implementation of governmental measures was reflected in the search behaviour of the general public [2]. The search for health-related information increased with the beginning of the SARS-CoV-2 pandemic [3]. Several studies have assessed the association between information search behaviour, knowledge and attitudes or behaviour related to infection control [4–6].

The information needs were encountered by a flood of information in newspapers, TV, internet, governmental websites, the Robert Koch Institute (RKI), social media and others. The quality of the provided information differed widely in relation to the nature of the reporting channel. For users, it is difficult to get access to the information which is for them relevant, understandable, trustworthy and reliable. Beside the intentional spread of misinformation and conspiracy theories, trustworthy sources may also offer misleading information due to inappropriate risk communication. For example, the communication of numbers of absolute cases without reference values can be alarming. This may lead to an overestimation of risks [7].

Not only laypersons but also politicians, healthcare professionals and health scientists may struggle with the flood of information and, simultaneously, the lack of knowledge and reliable data. Since the first outbreak of the new SARS-CoV-2, a lot of research has been initiated, resulting in a huge number of publications, reports, preprints and discussions of results. It is a major challenge to keep an overview and to assess the quality and reliability of published results. In addition, several questions still remain open. Therefore, decisions had and have to be made despite the uncertainty, and prior decisions may need to be revised. Under these conditions, it is challenging to communicate with patients or the general public, to involve them in decision-making processes or gain adherence to mandatory measures for infection control.

The COVID-19 Evidence Ecosystem (CEOsys) aims to address these challenges [8]. The CEOsys project is funded under a scheme issued by the Network of University Medicine (Nationales Forschungsnetzwerk der Universitätsmedizin (NUM)) by the Federal Ministry of Education and Research of Germany (Bundesministerium für Bildung und Forschung (BMBF)).

The idea of CEOsys is to provide “living” evidence syntheses, i.e. evidence that is constantly updated with the latest scientific results, directly feeding “living” medical guidelines and constantly disseminating information to the different target groups: clinicians, politicians and scientists as well as patients and the general population. The aim is to enhance informed decision-making based on the best available evidence.

An informed decision is based on relevant knowledge and is consistent with the patient’s values and preferences [9]. Most people prefer being involved in decision-making processes in healthcare [10]. In addition, the ethical rights [11] and the German act on patients’ rights [12] require the provision of understandable and comprehensive information and participation of patients in decision-making processes. Evidence-based health information is prerequisites for informed decision-making. Quality criteria for evidence-based health information have been widely described [13–16]. One important aspect is the involvement of the target group in the development process [13, 17]. This includes the assessment of the information needs and preferences of the target group, especially in such an unknown field like the SARS-CoV-2 pandemic. Therefore, surveys in the different target groups of CEOsys were conducted to assess their special needs [18]. This paper reports the result of the survey addressing the needs of the general population.

### Objective

To explore the public information needs and preferences in relation to COVID-19, and the barriers to accessing evidence-based information. The aim is to identify the relevant information topics with regard to COVID-19 and the SARS-CoV-2 pandemic and to assess which dissemination strategies and information formats are preferred by the general public. In addition, we explored the ability to appraise trustworthy information sources and understandable information formats.

## Methods

The reporting of this cross-sectional study follows the criteria of the Strengthening the Reporting of Observational Studies in Epidemiology (STROBE) Statement [19] and the Checklist for Reporting Results of Internet E-Surveys (CHERRIES) [20] (see Additional files 1 & 2).

### Recruitment and participants

We conducted an online survey among German adults registered in the HeReCa-online-panel (Health Related Beliefs and Health Care Experiences in Germany, https://www.medizin.uni-halle.de/hereca). The panel started in 2019 and up to now, participants in five federal states (Saxony-Anhalt, Berlin, Schleswig-Holstein, North Rhine-Westphalia and Baden-Wuerttemberg) have been recruited. In each federal state 14 to 15 communities or cities were chosen for the random selection of 10,000 citizens from local population registries, who were contacted via regular mail. From the initially contacted 50,000 citizens (10,000 per federal state), 3270 registered for the online panel. Together with the first questionnaire, they were sent information on the study and data protection measures via mail and were asked for their written informed consent. Registered members receive three or four questionnaires per year to obtain sufficient data from different regions of Germany on current discussions, public opinions or preferences related to health issues and policies. Participation is voluntary; members receive no incentives.

The invitations to our survey were sent by email to 927 registered panel members in the five federal states on December 17, 2020. The email contained the link to the questionnaire. To prevent or detect multiple entries from the same individual, all the participants logged in with a unique access code. Two reminders were sent out. The survey was closed on February 18, 2021.

### Questionnaire

The survey was set up in LimeSurvey [21]. The question formats were multiple-choice and open-ended. Participants could navigate between questions, revise answers and interrupt the survey as long as they had not finally submitted it. There was no “non-response” option, but answering the single items was not mandatory.

The survey started with a brief introduction on the CEOsys project, inviting participants to support the development of target group specific information on COVID-19. The questionnaire comprised nine items on nine pages in a fixed order. Items were divided into 25 sub-questions in total, partly only conditionally displayed based on previous responses (see Additional file 3 for the questionnaire). Socio-demographic data were not assessed in the current survey, as they were obtained in the survey directly following registration.

The first item assessed personal experiences with SARS-CoV-2 and/or COVID-19, since personal experiences may be the reason for a change in information needs and preferences.

The second item assessed the focus of interest on COVID-19 related topics. Participants were asked to select out of nine topics those most relevant regarding their everyday life (e.g., infection control or symptoms of COVID-19; at maximum three topics). Participants who selected ‘infection control’ could further select areas they were most interested in (e.g., infection control at schools or in nursing homes). All the participants could explain their selection and name aspects they were most interested in (open-ended question).

Four items assessed the preferred presentation and dissemination strategies. One multiple-choice question asked for the preferred media or channels for disseminating information (e.g., TV, newspaper, Facebook, or newsletter). The other three items asked for the preferred information formats (online and/or print, websites and/or documents for downloading, additional videos yes/no), information strategies (push/pull) and whether the participants would use a feedback option on an information webpage such as the CEOsys site.

Three items assessed barriers to accessing evidence-based information. One item was related to the risk communication in the media and related risk estimations. In the media, the communication of numbers referring to the SARS-CoV-2 pandemic in different countries is common. Especially at the beginning of the pandemic, the media presented the total numbers of newly infected people and deaths without reference values such as ‘infections per 100,000’. Only reference values allow a ranking of the different countries and a realistic risk estimation. In the questionnaire, we presented a table of European countries with the total numbers of infections and deaths only. We asked the participants to name the country most affected by the SARS-CoV-2 pandemic. They could choose one of the countries or the answer “I cannot tell with the given information”. In a second question, we asked for an estimation of the percentage of people in Germany who will have been infected with SARS-CoV-2 by the end of 2020. In the next item, we assessed how well-known different institutions providing health information are and whether they are rated as trustworthy. In addition, participants had to select criteria that were relevant for the appraisal of the trustworthiness of health information. In the last item, we assessed the perceived barriers to accessing evidence-based health information (multiple choice and open-ended).

A multi-professional team within the CEOsys network and the HeReCa panel developed and discussed the questionnaire. Parallel to that, the CEOsys working group set up surveys for other target groups (e.g. health professionals in intensive care and public health professionals). If possible, the items were adapted for use in the various surveys. We performed a pilot test of the questionnaire with undergraduate students in nursing science (*n* = 27, 3rd semester) which resulted in minor revisions only.

### Analyses

Data collection and management was carried out according to the General Data Protection Regulation. Data was stored on servers at the Martin Luther University. We included all data sets as long as answers to at least one item were provided.

Quantitative data were analysed descriptively. We used the statistical programme SPSS [22] to generate frequencies, percentages and mean values with standard deviations. References value for percentages is always N, the total number of participants including those who have not given any information. Due to the extension of the survey period into 2021, we performed a subgroup analysis of estimations of the percentage of people in Germany who will have been infected with SARS-CoV-2 by the end of 2020 provided in 2020 and 2021.

Two authors (AS, JL) carried out a content analysis to categorise the qualitative data from the open-ended questions. They summarized and paraphrased the answers and derived categories deductively (related to the questionnaire) and inductively (from the material).

## Results

We received 664 data sets, 28 were excluded (codes with double access and no or incomplete data), 636 could be included into the analyses. With 927 invited panel members, the response rate was 68.6%. Five hundred ninety-eight of the participants have completed the questionnaire. Reasons for non-participation were not assessable. Table 1 provides the participants’ characteristics.

**Table 1:** Characteristics of participants

Characteristics	Total* (*N* = 636)
**Sex, n (%)**	
Female	357 (56.2)
Male	244 (38.4)
Diverse	2 (0.3)
**Age, mean (range)**	
	52 (21-85)
**Marital status, n (%)**	
Single	145 (22.8)
Married	378 (59.4)
Divorced	55 (8.6)
Widowed	21 (3.3)
**Region, n (%)**	
Baden-Wuerttemberg	124 (19.5)
Berlin	172 (27.0)
North Rhine-Westphalia	99 (15.6)
Saxony-Anhalt	117 (18.4)
Schleswig-Holstein	124 (19.5)
**Nationality, n (%)**	
German	588 (92.5)
Other	12 (1.9)
**Migratory background**^**a**^** n (%)**	
	120 (18.9)
**School education, n (%)**	
> 10 years	413 (64.9)
10 years	154 (24.2)
< 10 years	29 (4.6)
Other	8 (1.3)
**Employment status, n (%)**	
Employed (full-time)	267 (42.0)
Employed (part-time)	93 (14.6)
Retired	141 (22.2)
Unemployed^b^	54 (8.5)
Other^c^	47 (7.4)
**Average net income per household per month, n (%)**	
< 1250 €	34 (5.3)
1250 to less than 1750 €	43 (6.8)
1750 to less than 2250 €	58 (9.1)
2250 to less than 3000 €	115 (18.1)
3000 to less than 4000 €	118 (18.6)
4000 to less than 5000 €	80 (12.6)
≥ 5000 €	97 (15.3)
No information	91 (14.3)
**Household size, n (%)**	
Living alone	113 (17.8)
2 persons	323 (50.8)
3 persons	78 (12.3)
4 persons	70 (11.0)
> 4 persons	15 (2.4)

^a^Foreign nationality and/or at least one parent not born in Germany; ^b^includes students; ^c^includes marginal employment and parental leave; *no information (n) 31-45

Fourteen participants (2.2%) reported that they had had a positive SARS-CoV-2 test (no information 0.6%). This corresponds to the proportion of registered infections in the population at the end of 2020 [1]. Only one out of the fourteen had to be treated in hospital. One hundred thirty-six participants (21.4%) reported that at least one family member or close friend had been tested positive, 24 (3.8%) that at least one related / close person had been treated in hospital and seven (1.1%) knew a person who had died of COVID-19. In total, 139 participants (21.9%) reported personal experiences with COVID-19 (no information 0.6%).

### Focus of interest on COVID-19 related topics

Most of the participants selected the topics *vaccination* (63.8%), *infection control* (52.0%), and *long-term effects* (47.8%) as the most relevant topics with regard to their everyday life (Table 2).

**Table 2:** Topics of interest

Most relevant topics related to the COVID-19 pandemic (selection of at maximum 3 topics)	Participants with personal experiences with COVID-19, n (%) (*N* = 139, no information 1)	Participants with no personal experiences / no information, n (%) (*N* = 497, no information 6)	Total, n (%) (*N* = 636, no information 7)
**Infection control**	73 (52.5)	258 (51.9)	331 (52.0)
*For which areas information on infection control is most relevant:*			
Hospitals & nursing homes	33 (23.7)	81 (16.3)	114 (17.9)
Medical practices	18 (12.9)	92 (18.5)	110 (17.3)
Sports	15 (10.8)	51 (10.3)	66 (10.4)
Working spaces	38 (27.3)	106 (21.3)	144 (22.6)
Schools	36 (25.9)	87 (17.5)	123 (19.3)
Public spaces & shopping centres	32 (23.0)	134 (27.0)	166 (26.1)
Restaurants	21 (15.1)	91 (18.3)	112 (17.6)
Theatres & museums	15 (10.8)	56 (11.3)	71 (11.2)
Other	10 (7.2)	30 (6.0)	40 (6.3)
**Symptoms**	25 (18.0)	105 (21.1)	130 (20.4)
**Test methods**	39 (28.1)	140 (28.2)	179 (28.1)
**Therapies**	51 (36.7)	164 (33.0)	215 (33.8)
**Intensive care**	10 (7.2)	26 (5.2)	36 (5.7)
**Palliative care**	3 (2.2)	13 (2.6)	16 (2.5)
**Vaccination**	87 (62.6)	319 (64.2)	406 (63.8)
**Mental health**	27 (19.4)	112 (22.5)	139 (21.9)
**Long-term effects**	73 (52.5)	231 (46.5)	304 (47.8)
**Other**	5 (3.6)	21 (4.2)	26 (4.1)

Five hundred sixteen participants explained their selection and named the aspects they were most interested in. We derived 11 categories from the qualitative analysis representing the topics of interest given in the questionnaire and further topics revealing information needs, concerns and preferences. The aspects participants were most interested in were assigned to these categories. In the following, we describe each category and subcategories if applicable.

1. In the category vaccination, we identified nine aspects participants were interested in. One was the effectiveness of the vaccines regarding immunity, the need for a booster vaccination, protection of those vaccinated and other persons (infectivity), and the relevance of mutations. A second aspect comprises risks and side effects, especially the risks for special groups (e.g. with pre-existing conditions, allergies, pregnancy), but also the risk for long-term effects or allergic reactions. Further aspects were the different kinds of vaccines (e.g. composition, comparison of effectivity and side effects, target groups, production and transport), recognition of harm through vaccination, strategies for vaccination (e.g. order of risk groups, time needed until the entire population is vaccinated, vaccination certificate), the vaccination offers (when, where and how), and the opportunity to work at the vaccination centres. The aspect of willingness to be vaccinated comprised comments on the individual decision and on the public willingness in relation to the likelihood of reaching herd immunity. Last aspect is the life after vaccination. Participants asked about the possibility of going back to normality and of living without fear of infection.
2. Infection control has nine subcategories: general, hospital and nursing homes, medical practices, sport, working spaces, schools, public spaces and shopping centres, restaurants, and theatres and museums. In *general*, participants were interested in a comparison between different settings - which places have a high/lower risk for infection? They asked about the pathways of infections, the effectivity of different measures for infection control (e.g., masks, distancing, ventilation, and partition walls), transparency of measures (suitable for everyday life), implementation and control of public measures, and personal measures (what can I do myself?). Further aspects are the possibility of attending public events, organization of “safe” family meetings, the handling of infected persons in the household, and travelling.
  Aspects regarding infection control in *hospitals and nursing homes* are the care of patients infected with COVID-19, the provision of protection material and sustainability (e.g., recycling of materials). The participants focused on the needs of special groups and were interested in the protection of risk groups without isolation, in visiting regulations and the compatibility with the need for support and in the implementation of measures for people with dementia (e.g., legal requirements).
  For medical practices we identified the following aspects: procedure for patients with symptoms of COVID-19, self-protection and/or attending appointments, tests and vaccination in medical practices, training for physicians to cope with psychological stress, and attitudes of physicians (role models).
  Regarding *sports*, the participants were interested in the question what kind of measures could enable sports, whether one should / could exercise with masks or how otherwise to protect oneself and how to act during the training. They pointed out the discrepancies between popular and professional sports. Further aspects were sports after an infection with SARS-CoV-2 and attending sports events as a visitor.
  One aspect regarding infection control in *working spaces* is the protection of employees, especially if contact cannot be avoided. Participants asked about safe contacts with clients / customers, business trips, personal protection measures, and tests for employees. Other aspects were employees’ rights, the regulations for working from home and compulsory presence. Participants mentioned psychological stress and sought advice on how to react to colleagues who did not follow protection measures.
  The participants asked about the options for keeping *schools* open, for safe classroom teaching, and about the priority for starting classroom teaching. They were interested in the risks and side effects of measures (e.g., lack of classroom teaching, but also the risk of wearing masks), and in the question whether / to what extent classroom teaching has an effect on the course of the pandemic (Differences between kinds of schools? Infectivity of children? What is known about infection chains in schools?). Further aspects were quarantine regulations and normal equipment in schools (e.g., sanitary facilities). Related to *schools* was higher education, e.g., universities. One aspect was the balance between infection control / protection and the mental health of students.
  Regarding *public spaces and shopping centres*, the participants called for carefulness between each other and asked about the possibility of providing more free spaces, especially for children. The unavoidable contacts in these spaces led to fear of infection.
  *Restaurants* were related to quality of life. The participants asked about reliable contact tracing, possibilities for safe services and support of restaurants’ owners (e.g., delivery service, outdoor), the infection risks despite hygiene concepts, and the effect of (indoor) restaurants on the course of the pandemic.
  Contact tracing and effects of closing down are also aspects that relate to *theatres and museums*. In addition, the participants asked about the risk of infection there in comparison to the risk in other areas (e.g., shopping, public transportation).
3. In the category long-term effects, the following questions and aspects were of interest: Which long term effects are already known and what is known about them (e.g., kind of effects, risk factors)? The participants meant the long-term effects of COVID-19 but also effects due to the vaccination. They asked about the prevalence, prevention, treatment, prognosis and diagnosis. Another aspect was the official recognition of long-COVID as an (chronical) illness (e.g., assumption of costs, occupational disability).
4. For therapies, there are three subcategories: treatment in general, intensive care, and palliative care. *General* aspects are treatment options (e.g., medication, what to do by myself?), harms and benefits of the medication, differences to the therapies of other respiratory diseases, influences of an early diagnosis, and prevention of a severe course of the disease (e.g., flu vaccination, strengthening of the immune system).
  With regard to intensive care, the participants asked about new findings and treatment options, the course of disease and long-term effects (e.g. psychological effects, course of disease in cases of dementia), artificial ventilation and alternatives, and for the protection of the staff.
  They also asked about the options for palliative care, the legal requirements and advanced care planning. A further aspect was anxiety; the fear of suffocating due to lack of oxygen, and the fear of exhausted resources in hospitals.
5. The category test methods includes the aspects differences between tests (e.g. reliability of tests, what is tested, procedure, optimisation of tests), access to tests (schedules, costs, addresses), test results (on short call, provision of results, procedure following a positive test result) and test strategies (e.g. test strategy for open schools, self-tests). A further question was whether an early diagnosis due to testing would influence the course of the disease.
6. In the category mental health, participants named different causes for increasing psychological stress (e.g., isolation, quarantine, unemployment, fear of infection, interventions in the daily life such as home office and schooling, alarmism in the media). They mentioned the effects on individuals and on the whole population, especially long-term effects because of the lockdown and isolation. They asked about how to deal with psychological stress (e.g., what to do by myself, resilience and coping strategies) and how to prevent depression, addiction or anxiety disorders. A further aspect is the association between mental and physical health (e.g., effect of anxiety on the immune system).
7. Regarding symptoms, the participants were interested in which symptoms may occur, their severity, frequency, which are typical, differences between groups and detailed information on single symptoms. They asked about how to recognize an infection with SARS-CoV-2 (e.g., reliable criteria for a diagnosis) and how to distinguish between COVID-19 and other diseases.
8. Participants were interested in the risk factors associated with a severe course of the disease.
9. The category strategies for pandemic control comprises several aspects such as implementation and control of measures for infection control, hygiene concepts and quarantine rules, close-downs and strategies for reopening, central vs. local management of measures, long-term measures and concepts (up to herd immunity), strategies for tests (e.g., mass tests for reopening of schools, provision of sufficient test sets) and for vaccination (e.g. allocation, reliefs in measures after vaccination). The participants were concerned about the compatibility of infection control and safety (especially for risk groups) and economics and public life.
10. The category infrastructure in healthcare has two subcategories. The first subcategory is the *need for information and support*. It comprises the desire of participants for support to cope with psychological stress and long-term effects (e.g., financial, for special groups, governmental offers), contact for further information and for telephone-counselling by physicians. There was a need for more reliable, understandable, up-to-date information (e.g., checklist of symptoms, information on long-term effects to reason for protection, information on tests, symptoms, treatment and vaccination, plausible explanation on the low risk for severe side effects after vaccination).
  The second subcategory is clinical care. The participants were concerned about the high-capacity utilization of hospitals and intensive care units, the (over-) load of the healthcare system and healthcare professionals, the differences between available resources, the possibility of triage, and the necessity of postponing operations and treatments. They pointed out how important good staffing, especially with nurses, was in order to maintain the proper functioning of healthcare systems.
11. Participants were interested in the current state of research and in new scientific findings. One important aspect was the development and approval of drugs (e.g., treatment options, antiviral medication). There should be funding for research on vaccination, long-term effects, chronic conditions due to vaccination and COVID, causal associations, molecular mechanisms and psychological stress. The participants wished for transparency in research, international and interdisciplinary cooperation in research and for the development of competencies, as well as the evaluation of current knowledge. For example, they expressed concern that the knowledge on the vaccines was still insufficient.

### Preferred presentation and dissemination strategies

**Fig. 1:**
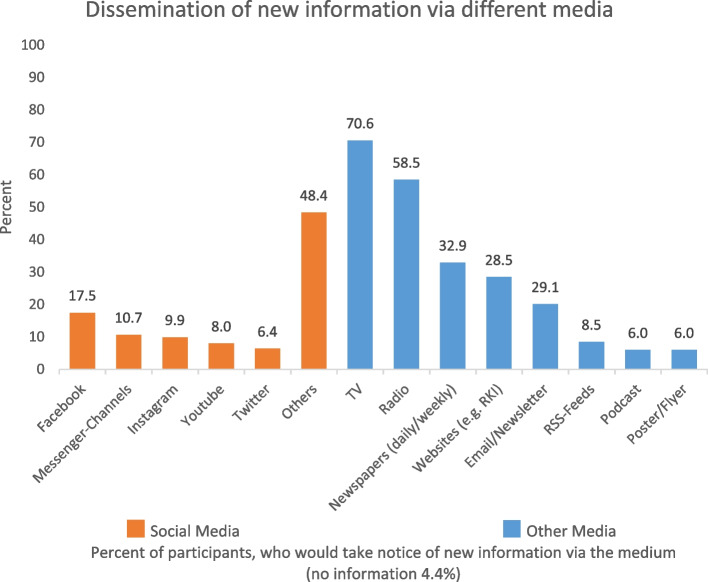
Dissemination of new information

Participants preferred using traditional media for becoming aware of new information about the pandemic (TV 70.6%; radio 58.5%; newspaper 32.7%) (Fig. 1). They also would notice online notifications on websites (28.5%) or via email/newsletter (20.1%). Social media or messenger channels were used to a lesser extent, Facebook if at all (17.5%). Those choosing newspapers were asked for the one they preferred. They named daily and weekly national newspapers (e.g.*, Süddeutsche*, *Welt*, *FAZ*, *Bild*, *Morgenpost* or *Zeit*), magazines such as *Stern*, *Spiegel* or *Focus* and a broad range of regional newspapers. The participants frequently selected the category “other”. We assume this was because of the two parts of the item. The first part asked for online community or social media and the second for campaigns or notifications in different media. At the end of the first part, the category “other” was offered. Most of the media participants named as “other” were options in the second part. In addition, they pointed out the corona warning apps.

In accordance with the result that traditional media are the preferred information sources, only 25.2% of the participants would prefer a push strategy (information is actively offered via email or social media). 67.1% chose the pull strategy with contents freely accessible without notifications (no information 7.7%).

58.0% of the participants stated that online information should be accessible on websites and documents for downloading should be provided. 29.1% of the participants would use information on websites only, 5.8% documents for downloading only (7.1% no information). 55.0% would like to have videos, illustrating the most important information (no videos 34.0%, no information 11.0%). The participants would rather not use a feedback option on an information website (yes/rather yes 41.1%; no/rather no 51.3%; no information 7.7%).

71.4% of the participants supported a print version of the online information for special groups such as elderly people who do not use online media, families or to all households (no information 11.6%). They proposed that general practitioners, nursing services, health insurance companies, health departments, regional authorities or newly created positions should provide that information. The participants suggested postal delivery of leaflets, supplements of newspapers, postings in showcases, houses, shops, restaurants, in public transport, hospitals or public institutions.

### Barriers to accessing evidence-based information

#### Risk communication in the media

We asked for the European country most affected by the SARS-CoV-2 pandemic. Only 7.5% of the participants gave the correct answer which was that they could not tell from the given information. More than 84% chose one of the countries (28.0% Italy with most deaths, 19.2% France with a high number of infections) and 7.9% provided no answer.

The mean estimation of the percentage of people in Germany that will have been tested positive for SARS-CoV-2 by the end of 2020 was 12.7% (SD 17.25; range 0-90%; *n* = 582). The RKI reported 2068 infections per 100,000 residents on December 31. Therefore, we defined an estimation between 1.5 and 3% as realistic. A realistic estimation was provided by 231 (36.3%) of the participants, a lower one by 48 (7.5%) and a higher estimation by 303 (47.6%). One hundred twenty-four participants provided estimations between > 3 and 10%, 82 between > 10 and 25%, and 97 more than 25%. Due to the extension of the survey period into 2021, we performed a subgroup analysis of estimations provided in 2020 and 2021. The mean estimation in 2020 (*n* = 362) was 11.1% (SD 16.05; range 0-90) and in 2021 (*n* = 207) it was 15.2% (SD 18.74; range 0-80).

#### Trustworthiness of information

**Fig. 2:**
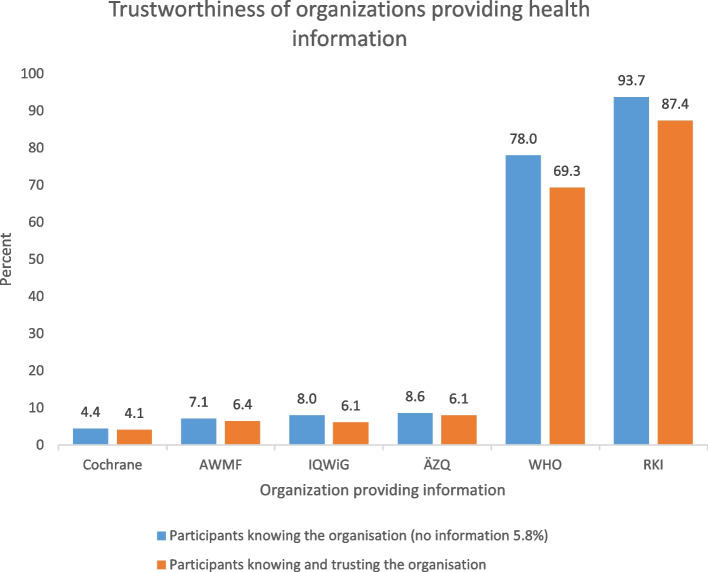
Trustworthiness of organizations providing health information. [Cochrane Collaboration; AWMF online – The portal of scientific medicine (patients’ guidelines); Patienten-Information.de (Medical Center for Quality in Medicine (ÄZQ)); gesundheitsinformation.de (Institute for Quality and Efficiency in Healthcare (IQWiG); World Health Organization (WHO); Robert Koch Institute (RKI)]

Almost all the participants knew about the RKI (93.7%) (Fig. 2). The World Health Organization (WHO) was also well-known (78.0%), but other organisations providing health information were hardly known (< 10%). A high proportion of the participants rated the organisations they knew as trustworthy (Fig. 2). In the free text comments, they named a wide range of other institutions providing health information: physicians, university hospitals, universities, non-university research institutes, international organisations (e.g. UNICEF, Red Cross), various political and administrative institutions (e.g. health departments, ministries), professional associations, medical societies, self-help organisations or patient initiatives, German Ethics Council, Federal Constitutional Court, different media and also alternative sources related to conspiracy theories.

Table 3 displays which quality criteria participants rated as relevant for the assessment of the trustworthiness of information. More than 50% of participants rated each of the criteria derived from the guideline evidence-based health information [13] as relevant, except the criterion “Funding and competing interests are reported” (36.5%). Participants considered information developed by experts (47.6%) or recommended by the physician (29.6%) also as trustworthy. Other quality criteria reported by participants in the free text comments were also related to meta-information provided in the multiple-choice list (e.g., meeting scientific standards, peer review, trustworthy editors, no advertisements, transparency), to presentation formats (wording, readability, structure, appropriate numerical and graphical display) or content (e.g., integration into existing knowledge, plausibility). Further, common sense was named and a scepticism towards all information.

**Table 3:** Criteria for trustworthy information

	Criteria to assess information as trustworthy (multiple selection possible)	Participants, n (%) (Total *N* = 636; no information 37)
Criteria for evidence-based health information [9]	Information sources / references are provided	539 (84.7)
	Development process is described (e.g. literature searches)	375 (59.0)
	Authors are named	354 (55.7)
	Information is up to date	330 (51.9)
	Funding and competing interests are reported	232 (36.5)
Other criteria	Information was developed by an expert	303 (47.6)
	Information was recommended by my physician	188 (29.6)
	Information is attractive and the design appears sound	51 (8.0)
	The information is widely used (at the top of Google search)	13 (2.0)
	Information was helpful for a relative or friend in a healthcare decision	13 (2.0)
	Other	33 (5.2)

#### Barriers to accessing evidence-based health information

17.5% of the participant considered none of the five given options as a barrier to accessing trustworthy, evidence-based health information. 48.3% selected one, 19.8% two and 8.4% more than two options. This resulted in 30.7% perceiving a lack of time as a barrier, 23.1% low experience, 22.2% uncertainty about how to get access, 23.9% complexity and difficulties in understanding, and 15.3% a lack of target group orientation (no information 6.1%). In addition, they reported on the one hand that it was too much information and on the other hand that information was not comprehensive and not reliable (e.g., conflicting or driven by interests). They reported the lack of accessibility to those with disabilities, the language (e.g., medical terms, foreign language) and costs as barriers. Some participants did not feel prepared enough to access information by themselves and would like personal contact. Others stated that there were no barriers or that they had no interest in evidence-based information.

One category from the qualitative analysis is conspiracy theories. This category was derived from the whole survey. One aspect was the question of how to handle such theories and the people believing in them. Participants asked how to react on, inform or persuade people who did not follow the instructions for infection control (e.g., not wearing masks). Other worried about relatives or close friends who had a different view on the SARS-CoV-2 pandemic. The second aspect was the loss of trust in media and information sources. There were statements such as that it did not matter who provided the information, all of the media or institutions would be lying, had to lie and, in consequence, that it would be better to use none of the information.

## Discussion

Our non-representative survey on the HeReCa online panel in 2020/21 revealed infection protection, vaccination and long-term effects as the most relevant topics for the general population in Germany. In general, information needs were rather high, comprising information on research, medical care structure, long-term strategies and risk assessments. Participants preferred rather traditional sources of information such as TV, radio and newspaper. Online information and social media had lower priority. In addition, participants preferred pull strategies and online information that could be downloaded. The majority would appreciate printed material, although all of them were members of the online panel. Trustworthy websites like those by the WHO und RKI were well known, however providers like the Institute of Quality and Efficiency in Health Care that provides evidence-based information were mostly unknown although the criteria of trustworthy sources of information were well identified, and barriers to accessing evidence-based information seemed to be fairly unknown.

In the CEOsys project, further surveys were conducted to assess the information needs and preferences of physicians, nurses and other target groups. The questionnaires included comparable items, adapted to the respective target group. One survey addressed the staff of intensive care units [18] and the other one the staff in in- and outpatient settings (under revision). The staff in intensive care units perceived a lack of experience with evidence syntheses and would like easily accessible information such as short summaries, algorithms and webinars. Nurses would also favour videos or podcasts. The RKI and medical societies were trusted information sources. Prioritized themes were the long-term effects of COVID-19, protection of the medical staff and the modes of ventilatory support [18]. Thus, the different results of the surveys support the aim of the CEOsys project to provide targeted information for specific groups or interested parties on the latest evidence synthesis using specific sources and media.

Our findings are in line with the results of Henrich et al. who conducted focus groups in Vancouver, Canada, in 2006 and 2007 to identify what information people want to receive and how they want to receive it in the event of a pandemic [23]. Study participants (students, parents and healthcare workers) wanted to know their risk of infection and how sick they could become if infected. They also wanted information on vaccines and drugs, which enables them to make decisions.

Prior studies surveyed information search behavior [3, 4, 23–26]. The results of three studies were in line with our findings. Führer et al. conducted a survey also addressing members of the HeReCa panel in three different federal states in Germany at an early stage of the SARS-CoV-2 epidemics and found that newspapers, radio and TV were the most important sources of information (45%) whereas websites and social media were only named by 23 and 3% respectively [26]. Meier et al. surveyed the three most frequently used sources to acquire information about the outbreak in Germany, the Netherlands and Italy and found that the information channels most frequently reported included television, newspapers, official health websites, and social media [25]. In addition, Henrich et al. revealed comparable information search behaviour in the focus group study in Vancouver, Canada [23]. The public preferred to receive the relevant information from family doctors, the Internet and schools. In addition, they acknowledged that the first information they received about a health crisis would come from mainstream media. This information was often perceived as untrustworthy [23]. Ali et al. conducted a representative survey in the USA where participants were asked about their use of 11 different COVID-19 information sources. Here the traditional media sources (television, radio, podcasts, or newspapers) were the largest sources of COVID-19 information (91.2%) [4]. In contrast to our findings, the survey conducted by Schäfer et al. that explored health interests and information search behaviour in students in Germany in 2019 and 2020 showed that 92% of the student sample preferred online sources [3]. In addition, the relevance of social media increased in students during the pandemic. More than half of the students stated that they used social media services, such as Facebook, Instagram, Snapchat, and Twitter for the search for information, which is also in contrast to our findings, as participants used social media to a lesser extent (17.5%). However, during the pandemic, the relevance of the classic offline mass media increased for students from 40 to 68%. In addition, interpersonal contacts with family members, friends and colleagues (81%) became even more important to the students as a source of information during the corona crisis [3]. Dadaczynski et al. investigated university students’ digital health literacy and web-based information search behaviours during the early stages of the SARS-CoV-2 pandemic in Germany [24]. Search engines, news portals, and websites of public bodies were most often used by the study participants as sources to search for information about COVID-19 and related issues. In addition, female students used social media and health portals more frequently, while male students used Wikipedia and other web-based encyclopaedias as well as YouTube more often [24]. The differences of the findings in comparison to our results are likely to be explained by the differences in age and educational background of the samples. Therefore, it seems urgent to consider the age of the target audience in addition to the different interested parties when providing information.

Eitze et al. surveyed trust in the RKI, the Federal Center for Health Education (*Bundeszentrale für gesundheitliche Aufklärung*, BZgA) and further governmental institutions and also physicians and hospitals [27]. Results showed that trust in the RKI and BZgA was generally high. These results are in line with our findings. In addition, Eitze et al. reported that the trust declined over the course of the pandemic (data collection 03/2020 - 08/2020). A survey in 2017 assessed the public’s familiarity with providers of online health information [28]. 24% were familiar with *Patienten-Information.de* and 26% with *gesundheitsinformation.de*. In our survey, the percentages were lower but the confidence in the institutions was high. In 2017, only around a third of those who knew the providers rated them as trustworthy. In contrast to our findings, Dadaczynski et al. reported that the greatest difficulties were found in assessing the reliability of health-related information (42.3%) and the ability to determine whether the information was written with a commercial interest in mind (38.9%). In addition, the use of social media was associated with a low ability to critically evaluate information, while the opposite was observed for the use of public websites [24]. Moreover, Okan et al. surveyed corona-related health literacy in a German sample where, although the overall level of health literacy was high, 47.8% reported having difficulties judging whether they could trust media information about COVID-19 [29].

We identified a category that we named “conspiracy theories”. Henrich et al. included parents known to be skeptical of, or opposed to, childhood vaccinations in their focus group study. In comparison to other parents, these “alternative” parents did not use traditional media sources for any of their health information. As well, these parents visited alternative health professionals rather than a general practitioner. Naturopaths and homeopaths were their preferred sources of information about a pandemic [23]. A survey in December 2020 showed that persons who agree with conspiracy theories were less willing to get vaccinated against COVID-19 [30]. A quarter of those who refused vaccination did not believe that an infection with SARS-CoV-2 might be harmful. Support of homeopathy and alternative medicine was also associated with a low willingness to get vaccinated. This association was also shown in the subgroup of parents [31]. In total, 47.6% of the parents would certainly or rather not let their children get vaccinated against COVID-19.

### Strengths and limitations

The thorough exploration of needs and preferences of target groups is essential for the development of complex interventions like the provision of information. The items had been thoroughly pilot tested. A large sample was involved and the response rate was fairly high. However, in comparison to the general German population [32], the participants were older, had a higher level of education and the proportion of persons with migration background was lower (about 19% vs. 25% in the general population). Especially the proportion of people between 40 and 60 years of age was higher in the survey participants than in the general population (about 43% vs. 28%). Nearly 65% of the participants fulfilled the entrance requirements for higher education; in the general population, the proportion was only 33.5% in 2019. The length of the study period (2 months) and the pandemic and political developments during this time may have influenced our results but we cannot say how.

### Implications and conclusions

Our survey has several implications. The results guide the distribution of information developed in CEOsys. Besides providing evidence-based information, strategies are needed to reach the target groups and make sure not to overlook existing barriers. Studies have shown associations of knowledge and behaviour during the pandemic. Schäfer et al. showed that a higher extent of a corona-related search for information in German students went along with higher compliance with recommendations aimed at containing the spread of the virus [3]. Therefore, reaching the target population is not just an end in itself but a key to achieving good compliance for the required measures. Stangier et al. reported that knowledge about SARS-CoV-2 predicted stronger preventive and adaptive behaviour but not stronger risk behaviour in students [33]. In addition, Ölcer et al. analysed social media posts and revealed an information pollution that also comprised misleading advice. Authors concluded that questioning the source of available information suggests the need and expectation for qualified information from scientists and related authorities such as departments or ministries responsible for health, and highlights the importance of presenting qualified information on social media [34]. These requests are in line with the *WHO guideline for emergency risk communication (ERC) policy and practice* (2017) which states that high quality information that is provided often and early and is adapted to the needs of the target group enables individuals to make choices and take actions to protect themselves, their families and communities from threatening health hazards [35].

Our survey also has implications for research. Taking into account the findings from the COVID-19 Snapshot Monitoring (COSMO) project [27], which surveyed people’s perception of the pandemic, and our own findings, the strategies for the distribution of information should be further evaluated with regard to efficacy.

In conclusion, people have extensive information needs regarding various aspects on SARS-CoV-2. The development of target group specific information and dissemination strategies are needed. In terms of pandemic preparedness, it would be useful to be able to draw on existing dissemination sources that are considered reliable in the eyes of the target audience.

## Supplementary Information

**Additional file 1.** STROBE checklist.

**Additional file 2.** CHERRIES.

**Additional file 3.** Questionnaire Public information needs and preferences on COVID-19.

## Data Availability

The datasets generated and analysed during the current study are available from the corresponding author on reasonable request. The datasets are in the German language.
