# Supplementary material for: Public information needs and preferences on COVID-19: a cross-sectional study
Source: BMC Public Health. 2023 Feb 27;23:394. doi: 10.1186/s12889-023-15131-x (PMC9969022; doi:10.1186/s12889-023-15131-x)
Supplement: Supplementary file 3 — Additional file 3. Questionnaire Public information needs and preferences on COVID-19. [file 12889_2023_15131_MOESM3_ESM.pdf]

Needs assessment CEOsys – Interviewing the public

To all participants:

Thank you for agreeing to take part in our survey.

The Corona pandemic is accompanied by a confusing flood of information. Despite this, or perhaps just because of this, it is often difficult to find reliable information. Many decisions, whether at the political level, in nursing homes, hospitals or even in the personal planning of everyday life, have to be made under considerable uncertainty.

Under the umbrella of the *Network of University Medicine,* the *COVID-19 Evidence-Ecosystem* project (CEOsys) was created. The objective of CEOsys is to set up a platform with reliable information on various issues concerning COVID 19. The information will be evidence-based, i.e. based on current scientific findings. For this, studies on the respective topic are systematically researched and evaluated with regard to their quality. The resulting statements will then be summarized systematically and prepared in an understandable manner for each target group. The CEOsys project is funded by the Federal Ministry for Education and Research.

Aim of this survey?

So that as many citizens as possible benefit from CEOsys, we want, on the one hand, to record with this survey the topics that are particularly important; but we also want to find out how you would most likely want to learn about new information and how such information should be provided.

By participating in this survey, you support the provision of comprehensible and reliable information. For this, a heartfelt thank you in advance!

Answering the questions will take you approx. 8 -10 minutes.

1. Personal experience with COVID-19:

1.1 Were you or are you yourself suffering from COVID-19?

Choice of answers (1 answer):

- No / I don’t know
- I was tested as positive, but had / have no symptoms
- I had / have only mild symptoms
- I had to be treated for COVID-19 in hospital
- I had to be treated COVID-19 in the Intensive Care Unit
- Not specified

1.2 Were one or more of your family or other persons related to you tested as positive but had no symptoms?

No / I don’t know   Yes

If yes: Number of people ___

1.3 Were one or more of your family or other persons related to you tested as positive but had only mild symptoms? (They did not have to be treated as in-patients in hospital.)

No / I don’t know   Yes

If yes: Number of people ___

1.4 Did one or more members of your family or other persons related to you have to be treated in hospital for COVID-19?

No / I don’t know   Yes

If yes: Number of people ___

1.5 Did one or more members of your family or other persons related to you have to be treated in an Intensive care Unit for COVID-19?

No / I don’t know   Yes

If yes: Number of people ___

1.6 Have one or more members of your family or other persons related to you died from COVID-19?

No / I don’t know   Yes

If yes: Number of people ___

2. Which topics related to Covid-19 are particularly relevant to your everyday life and should, in your opinion, be addressed in the CEOsys project?
   Please chose up to 3 topics that are particularly important to you.

Choice of answers (max. 3 answers):

- Measures to protect against infection with the corona virus SARS-CoV-2
- Symptoms
- Testing
- Treatment possibilities
- Care for COVID-19 Patients in the Intensive Care Unit
- Palliative care, aimed to relieve suffering and not to heal
- Vaccinations
- Mental health
- Long-term effects
- Others: [*free text*]

Depending on the choice, for each of the ticked topics:

You have chosen the topic … as being particularly relevant. Which aspects interest you most?

[*free text*]

Exception: If the first option „Measures to protect against infection" was ticked, a multiple choice menu would open:

Which aspects interest you most?

Selection (multiple choice)

- Hospitals and care homes
- Doctor's surgery
- Sport
- Place of work
- Schools
- Public places and shopping centers
- Gastronomy
- Theaters and museums
- Others: [free text]

Which aspects are you particularly interested in? [*free text*]

3. Risk communication in the media

Every day, data from John Hopkins University are presented in the media.

Figure with the following data was presented:

Corona virus cases worldwide December 15, 2020

Europe

**Coutry**	**Infected total**	**deaths**
Russia	2707945	47968
France	2391447	59072
Turkey	1898447	16881
UK	1888116	64908
Italy	1870576	65857
Spain	1762212	48401
Germany	1361039	22816
Poland	1147446	23309
Ukraine	925321	15792
Netherlands	628577	10168
Belgian	609211	18054
total	73094668	1627136

3.1 Which European country is affected most? (*1 answer*)

- (*Select European country from drop-down menu*) is affected most.
- Cannot tell on the basis of the information

3.2 Please estimate how many percent of the German population will have been tested as positive for Corona SARS-CoV-2 by the end of 2020.

Enter: ____%

4. How would you most likely become aware of new information??
   Choice of answers [Multiple choices possible]:

   Online Community or Social Media
   - Twitter
   - Facebook
   - Instagram
   - YouTube
   - Messenger Channel (z.B. Telegram)
   - Other: [*free text*]

   Campaigns or advertisements via:

   - Automatic newsfeed (RSS-Feed)
   - Email/Newsletter
   - Television
   - Radio
   - Podcasts
   - Daily/Weekly press
     If yes, which newspaper / journal: [*free text*]
   - Website of Robert-Koch-Institute, AWMF, Federal Ministry of Health etc.
   - Posters or flyers e.g. in doctors' surgeries or public facilities

5. In which kind of format should the health information be provided?

   5.1 When information is provided online, then it should …

   Choice of answers [*1 answer*]:

   - … be entirely digital, to be read directly on a website.
   - … be a PDF (printable) to be downloaded from a website.
   - … be able to be read directly digitally and downloaded as a PDF.

   5.2 In addition to texts I would like videos that explain the main points:
   Yes No

   5.3. When you think of people around you, shouldn't the information be available not only online but also in printed form?
   Yes No

   If "yes", do you have a suggestion as to where the information should be displayed or how it could be distributed?
   [*free text*]

6. Organizations that provide the information

6.1 Have you heard about the following organizations that provide the information?

- Cochrane Collaboration

Possible answers: Yes /No *If "yes" is ticked, the following question pops up:*

Do you consider the Cochrane Collaboration to be trustworthy?

Yes No

***The other organizations were checked along the same lines*:**

- AWMF online – The portal of scientific medicine (patients' guidelines)
- Patienten-Information.de (Medical Center for Quality in Medicine (ÄZQ))
- Gesundheitsinformation.de (Institute for Quality and Economy in Healthcare (IQWiG)
- Weltgesundheitsorganisation (WHO - *World Health Organization*)
- Robert Koch Institute (RKI)

6.2 Are there other organizations that you consider trustworthy? Please state: [*free text*]

7. How do you determine whether health information is trustworthy?

Choice of answers: [Multiple choices possible]:

- The source of the information is indicated (references)
- The information can be found at the top of a Google search, which means that it is widespread and used by a lot of people
- The authors are named
- The information is up-to-date
- It is stated how the preparation of the information was financed and possible interests of the authors are disclosed.
- The information was very helpful when one of my relatives had to made a decision concerning health
- The information was provided by an expert on the topic
- The procedure for creating the information is described (e.g. research of current scientific studies)
- The information is attractively designed and the design looks reputable
- The information was recommended by my doctor
- Others: [*free text*]

8. Which barriers complicate your access to trustworthy, *evidence-based information**?
    Choice of answers: [Multiple choices possible]:
    - I have too little time to bother about evidence-based information
    - I have no experience in dealing with evidence-based information
    - I am uncertain/don't know where or how I can access reliable evidence-based information
    - Evidence-based information is, from my point of view, very complex and difficult to understand
    - Evidence-based information is, from my point of view, not geared well to my target group (e.g. regarding foreknowledge, relevance)
    - Others: [*free text*]

**Evidence-based information* consists of current scientific findings. For this, a systematic search for studies on the particular subject is made and their quality is evaluated. The findings are summarized systematically and prepared for the respective target group in an understandable format.

9. Please let us know how you would prefer information to be provided.
    Choice of answers (1 answer):
    - Topics and content are presented to you directly; e.g. E-Mail or Social Media messages with links to the information ("Push Strategy")
    - Topics and content are freely available. How often you access them and how intensively you deal with the contents is entirely up to you. ("Pull Strategy").

10. Would you use the opportunity to give the CEOsys project members a feedback?
    Choice of answers (1 answer):
    - Yes, I would
    - Probably
    - Probably not
    - No, I wouldn't
